# Medical Learner Perspectives on Restorative Practices to Address Medical Racism

**DOI:** 10.1001/jamanetworkopen.2026.12459

**Published:** 2026-05-13

**Authors:** Crystal E. Brown, Almeda Jones, Benjamin Y. Hoisington, Arisa R. Marshall, Cyndy R. Snyder, Georgina D. Campelia, Bessie A. Young

**Affiliations:** 1 Cambia Palliative Care Center of Excellence at University of Washington Medicine, Seattle; 2 Division of Pulmonary, Critical Care, and Sleep Medicine, Department of Medicine, University of Washington, Seattle; 3 Department of Bioethics and Humanities, School of Medicine, University of Washington, Seattle; 4 University of Washington, School of Social Work, Seattle; 5 Department of Biobehavioral Nursing and Health Informatics, School of Nursing, University of Washington, Seattle; 6 Department of Family Medicine, Center for Health Workforce Studies, School of Medicine, University of Washington, Seattle; 7 Division of Nephrology, Department of Medicine, University of Washington, Seattle

## Abstract

**Importance:**

The use of restorative practices may be an alternative approach to responding to patients and families harmed by medical racism. Little research has explored its implementation in health care settings or the perspectives of residents and fellows (ie, medical learners) regarding its use in academic clinical settings.

**Objective:**

To elicit perspectives from medical learners on using restorative practices to address patient and family concerns about racism and other identity-based harms in the health care context.

**Design, Setting, and Participants:**

This qualitative study consisted of one-on-one semistructured interviews with residents and fellows between May and July 2024. Participants were invited from 76 adult and pediatric training programs affiliated with the University of Washington. The interview guide was developed by the study team, and interviews were conducted via videoconferencing and lasted approximately 30 minutes. Each participant received a $50 gift card as compensation.

**Main Outcomes and Measures:**

Medical learners’ perspectives and concerns about implementing restorative practices to address racism in health care settings. Thematic analysis was used to identify these perspectives. Interviews were conducted until thematic saturation was reached.

**Results:**

A total of 20 medical learners were interviewed. These participants had a mean (SD) age of 32.3 (3.2) years; 13 (65%) were women and 8 (40%) identified as Asian, 4 (20%) as Black, and 8 (40%) as White individuals. Eight participants (40%) had heard of restorative practices, mostly in the criminal justice and educational settings; 5 (25%) were confident in explaining what restorative practices entailed; and 2 (10%) had participated in restorative circles. In general, medical learners were supportive of using restorative practices to address medical racism and other harms experienced by patients and families. However, medical learners identified important concerns regarding preparation for this work (prework and intention setting), burden of participation (time barriers and personal and professional risks), and goals and outcomes (patient and family willingness, goals identification, and healing and harm mitigation).

**Conclusions and Relevance:**

This qualitative study found that medical learners were open to working with patients and families using restorative practices to address medical racism and other identity-based harms; these participants also identified additional concerns regarding implementation such as preparation, barriers and risks, and meaningful goals and outcomes. Future studies should investigate potential solutions to these concerns as well as include perspectives from patients and families to support evidence-based implementation.

## Key Points

**Question:**

What are the perspectives of residents and fellows (ie, medical learners) on the use of restorative practices to address medical racism?

**Findings:**

In this qualitative study of 20 medical learners, participants were supportive of using restorative practices to address racism and other intersectional harms experienced by patients and families in the health care setting. Medical learners also identified important considerations regarding the implementation of this approach: preparation, barriers and risks, and meaningful goals and outcomes.

**Meaning:**

The findings of this study suggest that restorative practices are feasible but warrant future studies that include perspectives from patients and families to support evidence-based implementation.

## Teaser

This qualitative study explores the use of restorative practices and perceived challenges associated with this approach among residents and fellows of adult and pediatric training programs at the University of Washington.

## Introduction

The detrimental effects of racism in health care settings are well known.^1,2,3,4,5,6^ However, practical frameworks for addressing its harms remain underexplored.^7,8,9^ Traditional institutional responses to racism often focus on policy rather than impact, risking insincerity and exacerbation of conflict.^10,11,12,13^ Restorative practices may be an alternative process for repairing harm, providing accountability, and facilitating healing.^10,14,15,16,17^ Restorative practices are based in Indigenous traditions in which involved parties voluntarily come together to define a path toward accountability and repair.^18^ These practices focus on the 5 Rs: acknowledging the damaged relationship; showing respect among all parties involved; the person who caused harm taking responsibility for their actions; repairing the harm, if possible; and reintegrating the person who caused harm back into their community.^8^

Guided by a trained facilitator, restorative practices are operationalized in restorative circles consisting of dialogue aimed at acknowledging and mitigating harm, achieving mutual understanding, and facilitating relational repair.^16,19,20^ Restorative circles are implemented in 3 different tiers: tier 1 works toward community building between individuals with commonalities, tier 2 addresses a harmful event, and tier 3 reintroduces the party that has caused harm back into their community.^21^ During the restorative circle, the facilitator allows each participant to share their perspective on an event or harm of concern, its impact, and ways to repair it.

Restorative practices are used in criminal justice and educational settings, with evidence suggesting these practices are associated with decreased psychological distress and increased trust and community building.^20,22,23,24,25^ Restorative practices are also used in academic settings, and the Association of American Medical Colleges supports its implementation to address learner harms.^26^ While restorative practices have been applied in health care settings to address harms and prioritize health care needs in marginalized communities, their implementation and evidence supporting their use are limited.^17,27,28^

A qualitative study was conducted of fully trained physicians in an academic setting who identified resources and processes aligned with restorative practices to address patient and family concerns about medical racism.^8,29^ However, residents and fellows (ie, medical learners) conduct the bulk of the clinical care and face-to-face interactions in academic settings and may be asked to participate in restorative practices. Concerns about power dynamics and potential disciplinary actions that may limit educational and career opportunities may make medical learners less willing to participate in restorative practices. On the other hand, there may be generational differences in perspectives about restorative practices, making medical learners more likely to participate. Hence, obtaining medical learners’ perspectives is important if restorative practices are implemented in academic clinical settings.

In this study, we aimed to elicit perspectives from medical learners on using restorative practices to address patient and family concerns about racism and other identity-based harms in the health care context. We anticipated that medical learners would be open to restorative practices and would identify additional insights into their implementation.

## Methods

### Study Design

Between May and July 2024, we performed a qualitative study using one-on-one semistructured interviews with medical learners in adult and pediatric residencies and fellowships. We used thematic analysis to identify medical learners’ perspectives, concerns, and challenges regarding implementing restorative practices to address racism within health care settings. The University of Washington Institutional Review Board deemed this study exempt from ethics review. Verbal consent was obtained from all participants. We followed the Consolidated Criteria for Reporting Qualitative Research (COREQ) reporting guideline.^30^

Our study team included 3 restorative practitioners (C.E.B., G.D.C., and B.A.Y.) and 2 clinical ethicists (C.E.B. and G.D.C.). Four of us (C.E.B., G.D.C., B.A.Y., and B.Y.H.) are directly involved in the education of medical learners. Members of this team are multiracial, multidisciplinary, and involved in various aspects of medical and nursing education as well as research into the inequities in health and health care. We acknowledge that researcher positionality—beliefs, understanding, and lived experiences related to race and racism—may shape the analyses and interpretation.^31,32^

### Participants

We recruited medical learners from residency and fellowship programs affiliated with the University of Washington. Participants were at least 18 years of age and were active in their clinical training program. One of us (C.E.B.) contacted program directors via email to provide study information and to obtain permission to approach their medical learners. A follow-up email was sent 1 week later if no response was received. Once permission was received from the program director, study information was disseminated by the program director, a program administrator, or a member of the study team. Both listservs and individual emails were used depending on the contact information provided to the study team. In addition to study information and a flyer, an email address for a research coordinator with no prior or future clinical obligations was included. Interested medical learners emailed the research coordinator to arrange a date and time for an online interview via secure videoconferencing (Zoom; Zoom Communications). Prior to the start of the interview, verbal consent was obtained for participation and audio recording. A $50 gift card was provided as compensation.

### Interview Guide

The interview guide (eAppendix in Supplement 1) was developed by the study team and refined to elicit medical learners’ experiences with patient and family concerns about medical racism and perspectives on using restorative practices to address these concerns. The restorative practices portion of the interview guide was informed by findings from a previous qualitative study of attending physicians^8,29^ and a study of a theoretical framework for acceptability of health care interventions.^33^ We included questions on medical learners’ understanding of restorative practices, the perceived amount of effort and risk in participating, and perceived effectiveness of these practices. Interviews were purposefully designed to be approximately 30 minutes in length to facilitate participation and accommodate medical learners’ busy schedules.

### Data Analysis

All semistructured interviews were conducted by a multiracial research coordinator with experience in interviewing marginalized patient populations and performing qualitative analyses (A.R.M.). Interviews were conducted until thematic saturation to ensure that breadth and depth of perspectives were captured from medical learners of various medical and surgical specialties as well as racial and ethnic backgrounds. Thematic saturation was defined as the point at which no new information was discovered.^31^ Race and ethnicity were self-reported by participants.

Interviews were audio-recorded with permission from participants, deidentified, and then transcribed. Interview transcripts were imported into and analyzed with a mixed-methods research software (Dedoose; Sociocultural Research Consultants, LLC).^34^ Three of us (A.R.M., C.E.B., and C.R.S.) reviewed the transcripts and, using open, inductive coding, produced a codebook. The preliminary codes were reviewed, edited, and approved by the study team (including A.J. and B.Y.H.). Three of us (C.E.B., A.J., and B.Y.H.) independently coded an additional set of 3 transcripts and then met to identify and reconcile coding discrepancies and refine existing or define new codes. A second set of 3 transcripts were subsequently coded independently, and then the codes were further refined and defined to create a final codebook. The remaining transcripts were coded independently, 80% of which underwent review by 2 or more study team members to ensure the codes were consistently applied. Any areas of disagreement were discussed until resolution was reached. Data analysis was performed using Dedoose, version 10.0.59 (Sociocultural Research Consultants, LLC).

## Results

We contacted the program directors for 76 out of 120 pediatric and adult medical and surgical residency and fellowship programs at the University of Washington. Of those contacted, 53 (70%) granted us permission to approach their medical learners; no response was received from the remaining programs. A total of 20 medical learners were interviewed. Participants had a mean (SD) age of 32.3 (3.2) years and included 13 (65%) who self-identified as women and 7 (35%) who self-identified as men; 3 participants declined to provide their age. One participant declined to be recorded, and the notes taken during this interview were coded. Eight participants (40%) self-identified as Asian, 4 (20%) as Black, and 8 (40%) as White. The medical learners’ specific training program is not disclosed, as such information could potentially be identifying.

Eight participants (40%) stated they had heard of restorative practices, mostly in the criminal justice and educational settings, but only 5 (25%) were confident in explaining what restorative practices entailed. Two medical learners (10%) had participated in restorative circles. One medical learner shared, “I used to be a teacher, so we implemented this [practice] sometimes in schools.” In general, medical learners were supportive of using restorative practices, as a participant noted, “The idea sounds really cool, to facilitate open and honest communication between patients and care teams in a way that mitigates the power differential.” However, participants identified important considerations about implementation, including sufficient preparation for the work; burden of participation, such as time barriers in addition to personal and professional risks; and desired goals and outcomes to ensure meaningfulness (Figure and Table).

**Figure.**
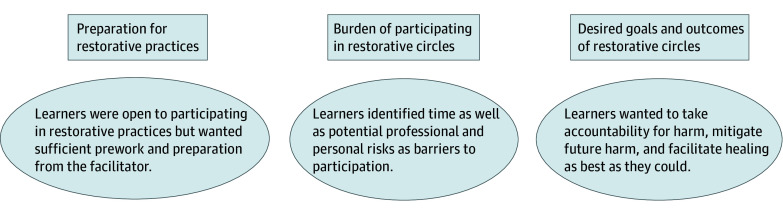
Schematic of Medical Learner Perspectives on Use of Restorative Practices to Address Medical Racism

**Table.** Medical Learner Perspectives on Implementing Restorative Practices

Themes and topics	Sample quotation
Preparation	
Prework	“The other would be being offered advanced notice of what this is, why I would be engaged in a conference like this, and being given the time to adequately reflect on a situation so that I feel that I’m able to speak truthfully and thoroughly about my experience, because, as I mentioned earlier, it can sometimes be hard to recollect things that have happened weeks or even days ago, especially without enough advance[d] warning to, really be able to reflect on the situation.”
Intention setting	“Knowing that there are people who are in your corner in some way and a clear stance that this is not to dissect what you did and to assign blame and do that. I think [that] would be really imperative because nobody wants to just go and be [accused] by people for a while and told ‘you know you’re racist.’”
Burden	
Time barrier	“I think time would probably be the biggest one. Are we blocking my clinic schedule? Am I coming in on my off time to do this? Like, how does this fit into the other things that I have to do, right?”
Personal and professional risks: confidentiality and disciplinary action	“What are the outcomes like…is somebody watching me to try to create a punishment? You don’t want to feel that. That’s not the point.”
Desired goals and outcomes	
Patient and family willingness	“Some people don’t want to see the [clinician], some people don’t want anything to do with the situation anymore. And forcing someone to sit with the people who have wronged them for an extended period of time and talk it out is difficult.”
Goal identification	“Is there a systemic change that can be gained from this? Is there some kind of, you know, repair to the patient? Health care system relationship that can be gained from this? Will there be legitimate benefit? Get some…metrics before we proceed instead of like, oh, you know, patient reported bias. Let’s have this conference.”
Healing and harm mitigation	“[H]aving that discussion before, before the meeting, itself with the facilitator to kind of get an idea of what they might be able to expect, in the meeting before going in, I think would be helpful. Maybe, perhaps getting a list of the topics to be discussed in the meeting or kind of the structure of the meeting, beforehand, I think would be helpful, just like for both parties, as much information, before the meeting as possible.”

### Prework and Intention Setting

In restorative practices, preparation consists of the interaction and prework done between the facilitator and involved parties prior to being brought together into a restorative circle. This preparation entails learning about the concerns that will be discussed and establishing a personal mindset that allows participants to both share their perspective and be receptive to others’ experiences. Medical learners understood the importance of preparation, noting their reliance on the facilitator to inform them about the events and to help with intention setting. One medical learner noted, “It would be important to set the intention at the beginning that it’s not necessarily about anyone doing anything right or wrong and that your intention might not have been to cause harm, but that’s what’s happened.” Another medical learner added that “being offered advanced notice of what this is, why I would be engaged in a conference like this, and being given the time to adequately reflect on a situation so that I feel that I’m able to speak truthfully and thoroughly about my experience…” was necessary (Table).

While medical learners were receptive to the experiences of patients and families, they also expected similar openness toward them. As a participant stated, “I think it’s important for everybody to know that everybody else is a human being and not a malevolent individual.”

### Time Barriers and Personal and Professional Risks

Medical learners identified multiple concerns and barriers to participating in restorative practices. There was unanimous concern about the amount of time required to meaningfully participate since medical learners were already overwhelmed by their clinical obligations. One medical learner shared, “It will be important for the department and leadership to provide time for you that’s not conflicting with other clinical duties, so you could be in that meeting without thinking about other things.” Another medical learner attributed nonparticipation to, “Just [lack of] time and conflicting responsibilities. I feel like most of us would be completely open to [restorative practices]. We just don’t know if it’d be feasible in practice.”

Medical learners were concerned about confidentiality and wanted to know who would be present in the restorative circle to prepare them, assess their psychological safety, and gauge their own willingness to attend. One medical learner shared, “I think having the involved parties themselves, like the patient and the [clinician], [is enough so they] can talk and understand each other better. Having a third party who is there just for a day and not involved in the interaction might not be as productive.” These concerns were closely related to unease about disciplinary actions that could compromise their reputation or future educational or employment opportunities. One medical learner wondered, “Is this a quality thing or is this more punitive? Is it something that the hospital is going to mark on my record?” Another medical learner expressed this uncertainty, “Is somebody watching me to try to create a punishment? You don’t want to feel that. The point is you hopefully learn and be better. Is there going to be a hospital lawyer there? Is the patient suing me or somebody? Some of those anxieties might make me hesitant.”

### Patient and Family Willingness, Goals Identification, and Healing and Harm Mitigation

Medical learners acknowledged the importance of establishing meaningful goals and outcomes prior to the restorative circle due to the potential risks involved for all parties. In fact, they were concerned about patient and family willingness to participate in restorative practices given their family and work obligations. Additionally, these participants were concerned about patient and family willingness to navigate persistent power differentials in spite of attempts to mitigate them. One medical learner shared, “I can’t help but wonder to what extent patients would want to go through all that,” while another stated, “From a patient side, I can envision this being a little challenging logistically.” As such, medical learners reported that identifying goals and desired outcomes were important. According to a participant, “You know, ‘Mr. Smith, is hoping to address this incident that happened and his and his family’s goals are to try to talk about a policy so that this doesn’t happen again.’ [In this way,] you have a sense of where the patient is coming from, what the goal is of that meeting specific to the patient.”

The most common desired outcomes shared by medical learners were healing and harm mitigation. A participant stated, “I think the biggest goal would be [to] get better communication skills in the future with whatever vulnerable population I’m working with, understanding that everybody’s not the same, but to become a little bit more sensitive in ways that I must be missing.” Medical learners hoped the restorative circle would promote taking accountability among themselves and other members of the care team, stating that hearing about the harm that occurred, even if unintentional, was a form of accountability. One medical learner shared, “I think it’s good reckoning to have to hear and reflect on that.” Medical learners hoped the restorative circle would bring healing to patients and families and repair damaged relationships. After reflecting on a situation in which they and their team were perceived by a family as racist, a participant shared, “I would definitely see [restorative practices] as being very helpful. Our team had the best intentions and just wanted to give the patient the best care that we could. We’re truly curious about what might have gone wrong so having a safe space to talk directly with the family would be extremely valuable.” Another medical learner’s goal was, “[p]roviding any sort of healing to the extent that I can to the harmed party and learning a thing or two about how to prevent these encounters in the future.”

## Discussion

In this qualitative study of medical learners’ perspectives on the use of restorative practices to address racism in health care settings, participants were open to restorative practices and recognized their potential to improve understanding of intent compared with impact of behaviors. Participants stressed the importance of prework and intention setting, time barriers, as well as personal and professional risks, and goals and desired outcomes that ensure meaningfulness. In contrast to a prior study with fully trained physicians,^8,29^ medical learners in the present study were concerned about repercussions that might adversely impact educational and professional opportunities. Both attending physicians (from the previous work) and medical learners reported the need for additional processes that are more conductive to healing and accountability. However, medical learners reported limited knowledge of restorative practices, suggesting an opportunity to improve knowledge and experience through tier 1 of the restorative circle for community building and moving on to tier 2 or tier 3 for addressing the harms.

Some precedence for restorative practices exists in Communication and Resolution Programs (CRPs). CRPs systematically respond to medical errors, recognizing that patients deserve and expect transparency and accountability.^35,36,37,38^ Restorative practices and CRPs are synergistic but distinct approaches in that they both respond to and support transparency through accountability, learning, and change.^39,40^ However, harm related to racism occurs within the context of broader marginalization and disparities in health care.^1,2,41^ Restorative practices may offer a unique ability to require accountability for the harms of medical racism while also recognizing that racist actions are viewed as an indictment of one’s moral character, resulting in shame, guilt, and defensive behaviors.^9^ Implementing an approach that concurrently demands accountability and addresses the associated shame may support healing and learning but requires a shift away from status-quo concepts of accountability and investigatory approaches that persist in health care institutions.^22,42^

While this study suggests a willingness from clinicians to participate in restorative practices, further studies are needed to examine how the barriers and concerns identified in this study can be meaningfully addressed. Medical learners brought up concerns about patient and family willingness to participate in restorative practices. Gathering perspectives from patients and families, particularly those who have experienced medical racism, is important to further assess the feasibility of implementing restorative practices. Patients and families may perceive a high level of personal risk but a low level of benefit and thus view such invitations as disingenuous. In addition, concerns persist about health care institutions acculturating and implementing practices originating from Indigenous communities, who continue to experience a high amount of interpersonal and systematic harms.^43,44^

### Limitations

There are limitations to our study. First, the design was a single-institution study, and the participants were small in number and predominantly consisted of White women. Medical students and medical learners from advanced practice programs were not included. While we had a nearly 70% response rate from program directors, the overall response rate from medical learners themselves was small, and there was variance in the ways potential participants were contacted; all of these factors limited the transferability of our findings. Medical learners in our study may have differing views from those who did not respond to our invitation, and program cultures may also be different between program directors who did and did not respond to requests.

## Conclusions

Medical learners interviewed for this study were open to using restorative practices to address medical racism and other identity-based harms with patients and families, noting that such practices may be a better approach than typical institutional investigational procedures. However, medical learners identified additional concerns such as preparation and intention setting, time barriers, as well as personal and professional risks, and meaningful goals and outcomes. Our findings suggest that implementing restorative practices may be feasible, but the concerns of clinicians as well as input from patients, families, and other health care workers will be critical to successful implementation and establishment as an evidenced-based practice.

## Notes

Supplement 1. **eAppendix.** Interview Guide

Supplement 2.
Data Sharing Statement
