# Supplementary material for: Medical Learner Perspectives on Restorative Practices to Address Medical Racism
Source: JAMA Netw Open. 2026 May 13;9(5):e2612459. doi: 10.1001/jamanetworkopen.2026.12459 (PMC13173387; doi:10.1001/jamanetworkopen.2026.12459)
Supplement: Supplement 1. — eAppendix. Interview Guide [file jamanetwopen-e2612459-s001.pdf]

Supplemental Online Content

Brown CE, Jones A, Hoisington BY, et al. Medical learner perspectives on restorative practices to address medical racism. *JAMA Netw Open*. 2026;9(5):e2612459
doi:10.1001/jamanetworkopen.2026.12459

eAppendix. Interview Guide

This supplemental material has been provided by the authors to give readers additional information about their work.

© 2026 Brown CE et al. *JAMA Network Open*

eAppendix. Interview Guide

Medical Learner Interview Guide Draft

Thank you for taking the time to talk with me. As described in the study materials, we are interested in learning about resident and fellow experiences with racism directed toward patients and their families, whether intentional or unintentional, in healthcare settings. We want to know your thoughts on how to both teach and support residents and fellows when patients or families have been harmed. We also want to gather your thoughts on programs, such a restorative justice, that promote healthcare worker responsibility and accountability when patients and families have been harmed. In a recent study we conducted with Black patients suggests that some patients want to discuss concerns about racism in clinical settings but can detect that a healthcare worker feels uncomfortable or that their concerns are too quickly dismissed. We are exploring ways to improve communication and participation in programs based on restorative justice but first want to gather information on potential concerns and barriers to your participation.

Suggested probes to encourage and explore information provided by participants:
"Tell me more about that."
"I'm not sure I quite understood – please explain XXX a bit more."
"That is quite interesting— what more can you tell me?"

Racism in healthcare settings
First, we're going to talk about racism in healthcare settings. Can you think of a time when a patient you were caring for or a family member was concerned that they or their loved one was experiencing racism or that racism was affecting their care? This could also include discrimination, feelings about bias, and subsequent mistrust from that.

- Would you be willing to share more about what happened?
- What was your involvement in that patient's care and what was your proximity to their concerns?
- Were these concerns reported anywhere? To whom or through what mechanisms?
- What happened afterward? What consequences occurred as a result of these actions?
- If you were offered an opportunity to meet with the [the patient and family] to discuss these concerns in specific, would you want to meet with them? Why or why not?

Scenarios
I'd like to present some scenarios to you in which racial tension is an important contextual feature of an interaction between a clinician and a patient. These are scenarios are adapted from interviews with Black patients with serious illness from a recent study we completed.

<u>Scenario 1:</u> Mr. Evans is a 64-year-old Black man with metastatic cancer. He is upset and feels that his pain is not being properly treated. He also thinks other treatment modalities are being withheld from him though he cannot say what exactly. He believes he is being mistreated because he is Black and doesn't have a lot of money. He wants to talk to his doctors more about this, but every time he brings this up, he feels his treatment team is too quick to try to reassure him. Mr. Evans has recently completed the PRISM program and is ready to speak with his team more frankly about his medical treatment, including talking about whether or not race or money is

© 2026 Brown CE et al. *JAMA Network Open*

playing a role in what the team decides to give him. He is worried about appearing as an angry Black man, especially since his treating team is made up of all White physicians.

- What's your first reaction when you hear this? What comes to mind?
- Have you been in a situation like this before?
    - How did you navigate it?
    - Was there anything that you wish you did differently?
    - What, if any, resources do you wish you had to help navigate this situation?

<u>Scenario 2:</u> Mrs. Johnson is a 59-year-old Black woman with *[provider specialty specific diagnosis].* She has been admitted multiple times for COPD exacerbations. Each time she is admitted, her care team attempts to discuss her goals of care and whether or not she would like to be intubated. Mrs. Johnson wants to be intubated and has always wanted CPR and wondering doctors continue to ask this question because they do not want to her to live because she is Black and has a history of using illicit substances in the past. While she is curious about this, she doesn't ask about it because she is afraid that her doctors and nurses will retaliate against her by withholding more treatment.

- What's your first reaction when you hear this? What comes to mind?
- Have you been in a situation like this before?
    - How did you navigate it?
    - Was there anything that you wish you did differently?
    - What, if any, resources do you wish you had to help navigate this situation?

Learning and receiving feedback
We are thinking of ways to teach HCWs about the harms patients and families experience from healthcare racism and bias. In particular we're interested in learning from residents and fellows since you all do the lion's share of the work, but are also the most vulnerable.

- If a patient or family member thought you were providing biased care, who would you want to hear this from? Why?
- Who would you <u>not</u> want to hear from and why?
- Could you tell me more about other expectations you have or what you envision about being told about concerns in the care you are providing?
- What kind of follow up would you expect after receiving feedback about your care? From whom?
- Residents and fellow are part of a union. What role do you see the union playing while you are receiving this feedback? What about afterward?

Restorative justice
Restorative justice circles are structured processes based in peacekeeping Indigenous practices that allow community members to voluntarily come together to address harmful behavior with a path toward accountability and repair. You might have heard of restorative justice circles as they have been used in different settings including schools, correctional and mental health facilities, and foster homes. Rather than focusing on policies or rules that have been broken, restorative justice circles instead identify and center those who have been harmed and what must be done to repair the harm. Instead of formal investigatory processes that focus on determining if an alleged offender acted unlawfully or otherwise violated a rule or policy, restorative justice circles responds to reports of bias by directly focusing on impacted persons and what must be done to repair the harm. These meetings are supported by facilitator who has received special training.

© 2026 Brown CE et al. *JAMA Network Open*

Prior knowledge or experience

- Have you heard of restorative justice circles before?
- *If yes*: What do you know about them? What is your experience with them?
- *If no*: Restorative justice circles are XXX. After hearing this, what are your initial thoughts about them?

We are interested in using restorative justice circles in the healthcare setting to facilitate meetings between patients and families and healthcare workers that have harmed them. These meetings would be mediated by a facilitator who has special training to lead these types of meetings. This facilitator would be using a guide or protocol to ensure that meetings are run fairly.

- Does this sound like something you might be interested in? Why or why not?

Hopes and concerns about participating in restorative justice circles

- If you were to participate in a meeting such as this, what might be some goals you would have for yourself?
- What would be important to you to have happen at the meeting?
  - What would you want to see happen *during* this meeting?
  - What would you want to see happen *after* this meeting?
- What are some of your concerns about participating in a meeting such as this?
  - What would you want to see done *before* the meeting in order to feel safe?
  - What would you want to see done *during* the meeting in order to feel safe?
- What can the facilitator mediating this meeting do to make sure you feel safe?
- If you were offered to the opportunity to participate in a restorative justice circle, what would be a barrier that would keep you from participating in one?
- Similarly, if you were offered the opportunity to participate in a restorative justice circle, what would encourage your participation?
- Who would you expect to be there at the RJ circle? Who would you not want to be present at the circle?
- What role do you see the union playing if you were asked to participate in a RJ circle?
- What kind of follow up would you expect to see from the facilitator or the hospital after participating in a restorative justice circle?

Wrapping up

- Think back to the experience that you shared with me at the start of this interview or the scenarios we discussed. How would a restorative justice circle have been helpful after experiencing that situation?
- Do you have any other thoughts that you would like to tell me?

Thank you for participating in this interview. I again want to assure you that your responses are confidential and that no one at UW Medicine will be made aware of your responses in this interview.

© 2026 Brown CE et al. *JAMA Network Open*
