# Supplementary material for: Medical Learner Perspectives on Restorative Practices to Address Medical Racism
Source: JAMA Netw Open. 2026 May 13;9(5):e2612459. doi: 10.1001/jamanetworkopen.2026.12459 (PMC13173387; doi:10.1001/jamanetworkopen.2026.12459)
Supplement: Supplement 2. — Data Sharing Statement [file jamanetwopen-e2612459-s002.pdf]

Data Sharing Statement

Brown. Medical Learner Perspectives on Restorative Practices to Address Medical Racism. *JAMA Netw Open*. Published May 13, 2026. doi:10.1001/jamanetworkopen.2026.12459

Data
Data available: No
